# The beating heart: artificial intelligence for cardiovascular application in the clinic

**DOI:** 10.1007/s10334-024-01180-9

**Published:** 2024-06-22

**Authors:** Manuel Villegas-Martinez, Victor de Villedon de Naide, Vivek Muthurangu, Aurélien Bustin

**Affiliations:** 1 IHU LIRYC, Electrophysiology and Heart Modeling Institute, Hôpital Xavier Arnozan, Université de Bordeaux–INSERM U1045, Avenue du Haut Lévêque, 33604 Pessac, France; 2 Department of Cardiovascular Imaging, Hôpital Cardiologique du Haut-Lévêque, CHU de Bordeaux, Avenue de Magellan, 33604 Pessac, France; 3 Center for Cardiovascular Imaging, UCL Institute of Cardiovascular Science, University College London, London, WC1N 1EH UK; 4 Department of Diagnostic and Interventional Radiology, Lausanne University Hospital and University of Lausanne, Lausanne, Switzerland

**Keywords:** Artificial intelligence, Cardiac, Magnetic resonance imaging, Heart, Deep learning

## Abstract

Artificial intelligence (AI) integration in cardiac magnetic resonance imaging presents new and exciting avenues for advancing patient care, automating post-processing tasks, and enhancing diagnostic precision and outcomes. The use of AI significantly streamlines the examination workflow through the reduction of acquisition and postprocessing durations, coupled with the automation of scan planning and acquisition parameters selection. This has led to a notable improvement in examination workflow efficiency, a reduction in operator variability, and an enhancement in overall image quality. Importantly, AI unlocks new possibilities to achieve spatial resolutions that were previously unattainable in patients. Furthermore, the potential for low-dose and contrast-agent-free imaging represents a stride toward safer and more patient-friendly diagnostic procedures. Beyond these benefits, AI facilitates precise risk stratification and prognosis evaluation by adeptly analysing extensive datasets. This comprehensive review article explores recent applications of AI in the realm of cardiac magnetic resonance imaging, offering insights into its transformative potential in the field.

## Introduction

**Fig. 1:**
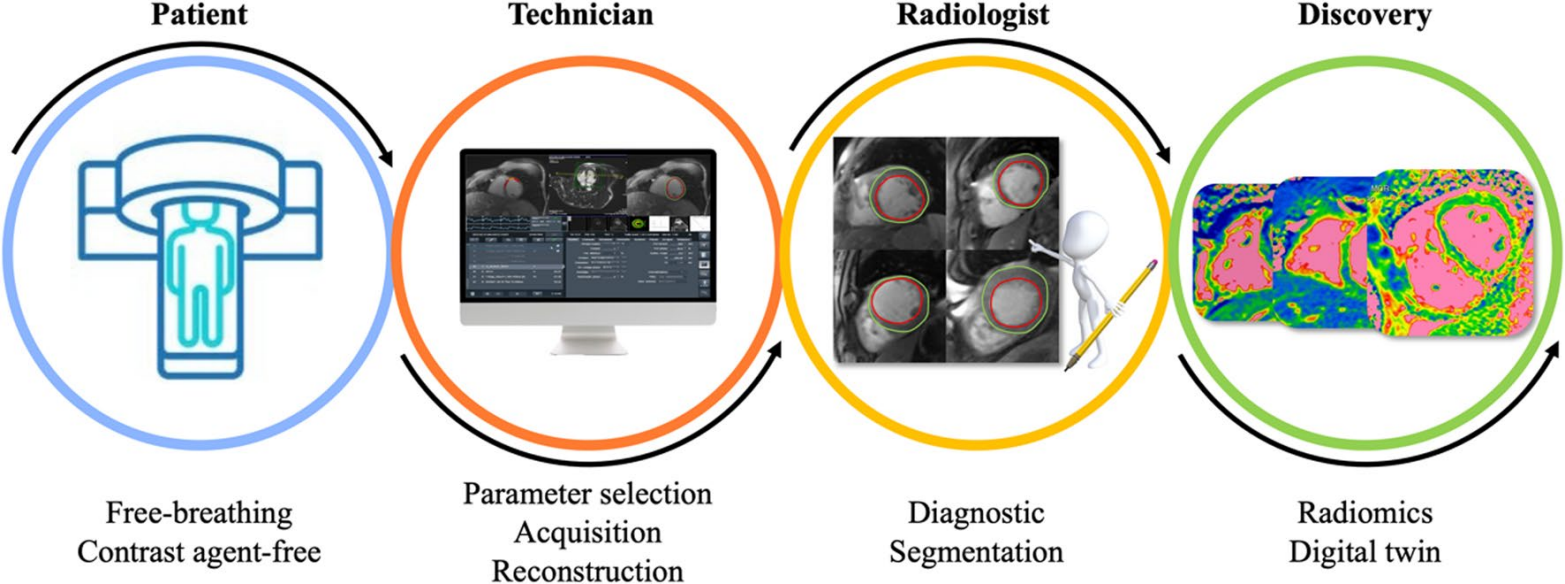
Identified fields that artificial intelligence has the potential to impact in cardiovascular magnetic resonance imaging

Cardiovascular magnetic resonance imaging (CMR) has gained widespread acceptance for its non-invasive assessment of cardiac anatomy and function, serving as a valuable tool in cardiovascular diagnosis [1–3]. However, limitations have been found in acquisition, post-processing, interpretation, and prediction outcomes [4]. Recent advancements in artificial learning (AI), particularly in artificial neural networks known as deep learning (DL), have shown their potential in overcoming many of these challenges, by automating post-processing and improving diagnosis and outcomes [5–7]. These breakthroughs, fueled by access to extensive data, user-friendly software frameworks, and enhanced computing power, have resulted in significant interest in AI from the cardiovascular community. Presently, these models represent the cutting-edge approach across various domains, including computer vision, language modeling, and robotics. In the realm of healthcare, the substantial volume of data generated and captured by providers contains valuable signals that traditional analysis methods struggle to process. ML has emerged as a powerful solution, facilitating the integration, analysis, and prediction of outcomes based on large, diverse data sets. In the context of cardiovascular imaging, DL algorithms enhance radiologists' workflow by reducing acquisition and post-processing time, improving image quality, and enhancing examination accuracy (Fig. 1).

Furthermore, AI aids in precise risk stratification and prognosis evaluation by efficiently analysing extensive data sets. The focus of this review is to provide an overview of the latest AI applications in CMR, emphasizing the prognostic value and the promising future of this potent yet relatively undiscovered technology.

## Patient and technician sides

One primary application of AI in cardiovascular imaging lies in optimizing the CMR examination process, benefiting both patients and technicians with quicker, streamlined scanning. For patients, this means reduced time spent inside the scanner, while technicians can attend to more patients. Currently, there are two main obstacles to fast efficient scanning: (i) scan planning/parameter selection and (ii) scan acquisition/reconstruction time. In this section, we will discuss how AI can be used to speed up both of these aspects of the CMR exam.

### Automated planning and parameters selection

Technicians play a crucial role in the field of CMR, being essential for the acquisition of high-quality images, which are crucial for diagnosis and prognosis. Their expertise in scan planning and in fine-tuning specific parameters significantly impacts image quality. However, current practices often involve manual and iterative parameter selection, leading to potential variations within and between magnetic resonance (MR) technicians, while increasing workload [8]. In this scenario, AI has the potential to assist technicians by improving the accuracy, reproducibility, and precision of examinations [9].

Traditionally, precise slice geometry prescription has been achieved through multiple scout scans involving breath-holding and manual adjustment of scan planes by a technologist. This process adds extra scan time, discomfort for the patient, and complexity to the workflow. Consequently, various studies have sought to automate these localization tasks, as well as other important tasks, such as shimming. Edalati et al. [10] utilized two AI-based techniques to automate cardiac planning (EasyScan) and shimming. EasyScan is based on a deep learning regression network for automatic slice alignment, and it was able to minimize technician-dependency and reduce examination time by two minutes. AI-based shimming provided an enhanced and more uniform B0 magnetic field homogeneity and a 12.49% higher signal-to-noise ratio. The results showed that AI could facilitate and accelerate the clinical workflow, minimize technical complexity, and, hence, improve patient tolerance.

However, setting scan planes is not the only responsibility of MR technicians; they must also set several parameters related to cardiac gating and imaging contrast.

#### Cardiac resting period detection

The majority of CMR techniques rely on electrocardiographic (ECG) gating to synchronize the pulse sequence with cardiac motion [11] and most data are acquired during the diastolic quiescence period to minimize motion-induced artifacts. The selection of this resting period currently relies on visual identification which can have a potential negative impact on scan reproducibility [12].

**Fig. 2:**
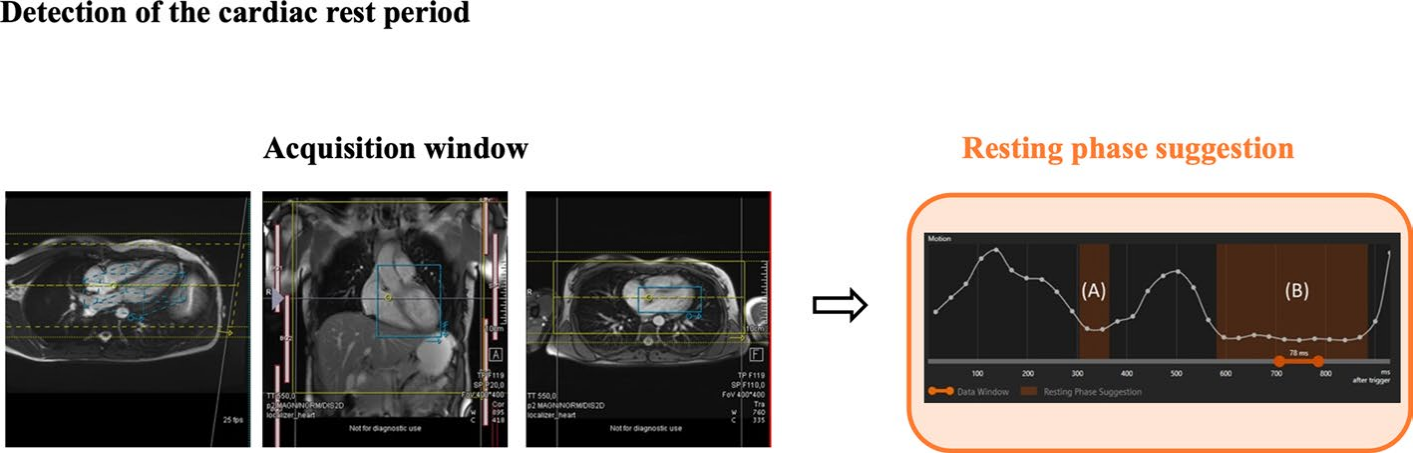
Automatic detection of trigger delay and acquisition window for whole-heart coronary magnetic resonance angiography imaging. A and B represent two different phase suggestions. The position of the coronal imaging slab (in yellow) and isotropic image-navigator box (in blue) are also determined (adapted with permission from Wood et al. [12])

In response to this challenge, Wood et al. [12] proposed a DL software designed for automated detection of the cardiac resting period. The algorithm localizes the right coronary artery in four-chamber cine images through segmentation, employing a U-Net, or landmark detection, utilizing a 3D-DenseNet. Subsequently, a trigger delay and an acquisition window targeting mid-diastole are calculated and incorporated into a high-resolution 3D whole-heart coronary magnetic resonance angiography (CMRA) acquisition. This methodology was able to determine the trigger delay one minute faster than the technician (106 ± 38.0 s vs. 168 ± 39.2 s, *p* < 0.01, 95% CI of difference 25.5–98.1 s) (Fig. 2). Another approach, proposed by Huang et al. [13], involved automatic identification of the cardiac resting period using conventional cine balanced steady-state free precession rapid free-breathing and the standard deviation method. This method enables the identification of end-systolic and mid-diastolic times from a cine image.

#### Inversion time selection in late gadolinium enhancement imaging

In current practice, another parameter requiring manual selection is the optimal inversion time (TI), which is essential for suppressing normal myocardial signal to assess myocardial fibrosis in late gadolinium enhancement (LGE) CMR [14]. This parameter is obtained after visual contrast selection on a TI-scout sequence, which collects multiple images with varying TIs. Due to its impact on detecting and delineating myocardial scar, it can significantly affect the diagnosis and prognosis value of the exam. For this reason, AI has been employed in various ways to assist in this selection process.

**Fig. 3:**
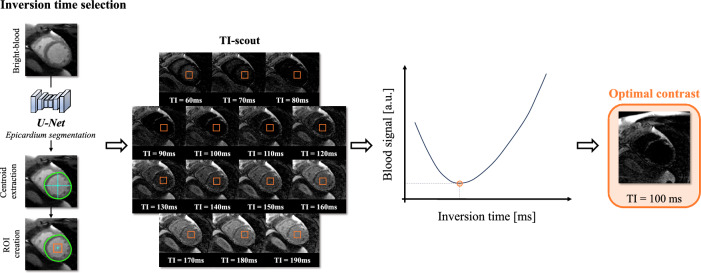
Automatic inversion time selection for LGE imaging. The algorithm detects epicardium contours, from which the centroid of the segmentation is extracted. Subsequently, boxes of empirically selected size (orange squares in figure) are created around the centroid and the signal intensity is extracted from the pixels in it

Bahrami et al. [15] utilized spatial and temporal imaging characteristics of TI images, using long–short-term memory convolutional neural network (CNN) structures, inputting a window of four different TIs to predict the optimal one from a bright-blood inversion recovery LGE sequence. The predictions made by the model closely matched expert annotations in 83% of the cases. Other studies obtained similar good results finding the optimal TI, by analysing the mean signal in segmented regions [16, 17]. Maillot et al. [18] presented an image-based algorithm for semi-automated TI selection from black-blood LGE imaging [19]. This algorithm uses the positioned shim box which contains pixels from the blood pool and myocardium. The optimal TI is determined by the TI image with the darkest signal within a defined region of interest, assessed using a simple threshold-based method. This approach showed good reproducibility, ease of implementation, time efficiency, and accuracy, with an agreement between the automated algorithm and the experts of 0.73 (Fleiss kappa coefficient) in 120 patients. However, the effectiveness of this technology relies on the accurate positioning of the shim box by the MR technician. This research was extended by de Villedon de Naide et al. [20], who proposed to fully automate TI selection on joint bright- and black-blood LGE imaging [21, 22]. This approach replaces the shim box dependence from the above technique with a U-Net-based detection of the epicardium on bright-blood images, making TI selection for black-blood LGE imaging fully automated (Fig. 3). It is worth noting that with phase-sensitive inversion recovery (PSIR) imaging, which restores the signal polarity, the need for a TI-scout sequence is obviated, thus eliminating the need for TI optimization [14].

#### Future perspectives

The automation of these parameter tuning tasks represents a substantial advancement in reducing technicians’ workload and reducing scan quality variability. Importantly, achieving time-efficient image acquisition without compromising quality can enhance patient comfort by reducing scan durations. Nevertheless, careful consideration must be taken regarding what tasks should and should not be automated. Maintaining human contact with the patient is a critical factor for patient compliance [23]. In addition to their technical duties, MR technicians play a crucial role in communicating with patients and ensuring their comfort throughout the imaging procedure.

### Acquisition and reconstruction

Another area where significant work is focused is in accelerating CMR procedures by reducing acquisition and reconstruction times. The need for images with high temporal and spatial resolution, and in some cases, whole-heart coverage often leads to long acquisition periods. In addition, the presence of both cardiac and respiratory-induced motion further complicates and extends the scanning duration [24]. Current state-of-the-art methods that address these challenges include non-Cartesian imaging, parallel imaging, and compressed sensing (CS).

In the context of CMR, acceleration exploits both multi-channel acquisition and redundancy present in spatiotemporal data. By incorporating non-Cartesian trajectories, significant acceleration (> fivefold) can be achieved in both 2D and 3D applications [24]. This undersampling does not compromise the accuracy or precision in estimating clinically relevant cardiovascular parameters [25, 26]. However, methods such as CS-based reconstructions are hindered by lengthy scan times. In addition, the choice of the sparsity representation and the tuning of the corresponding reconstruction parameters directly impact the performance of the reconstruction. Consequently, there has been a growing interest in the use of AI for reconstruction, aiming to accelerate computation and enhance image quality. ML has revolutionized the image reconstruction domain and is swiftly establishing itself as the forefront technology. DL-based reconstruction algorithms have surpassed conventional methods, producing more precise image reconstructions and facilitating the use of higher undersampling factors [27–29]. In this section, we will review the use of AI to accelerate 2D cine and 3D imaging.

#### Cine imaging

Accurate cine imaging is part of almost all CMR protocols, as it is used to reliably assess cardiac function. To expedite this process, Kofler et al. [30] suggested a method for recovering undersampled 2D radial cine CMR by training a modified 2D U-Net. This approach demonstrated similar image quality to CS, with quicker reconstruction times and small amounts of training data requirements. In addition, imaging acceleration has also enabled the acquisition of 3D cine left ventricular (LV) coverage within a single breath-hold. Despite showing good agreement in LV function compared to the gold-standard 2D cine, this approach is constrained by limitations in resolution and reconstruction duration due to anisotropy [31].

Another approach to cine imaging that benefits from high levels of acceleration is real-time imaging. Real-time imaging enables assessment of dynamic changes without relying on ECG gating, which means that it can be acquired during free breathing. This is particularly useful in children and sicker patients who struggle with breath-holding. However, real‐time imaging requires significant data undersampling to ensure adequate spatio-temporal resolution.

Previous non-Cartesian real-time methods have shown effectiveness but are limited by long reconstruction times. Hauptmann et al. [32] proposed the use of a U-Net to reduce undersampling artifacts for 2D golden-angle radial cardiac cine MRI. This method significantly reduced reconstruction time, being at least five times faster than traditional CS reconstruction while also delivering superior image quality. The results obtained proved that 3D CNNs could efficiently map entire undersampled 2D + time sequences. It should be noted that the presented methods are applied in post-processing. Using validation data consisting of coil-combined magnitude images rather than multi-coil complex k-space data implies that the models do not learn the full reconstruction procedure and thus do not take advantage of coil sensitivity encoding inherent in parallel imaging [24]. As a result, there is a growing interest in reconstruction methods that combine iterative methods with DL, as they include data consistency and may provide better reconstruction accuracy [33, 34].

Real-time imaging is particularly beneficial in cardiac procedures, such as MRI-guided catheterization. However, they must have low-latency reconstructions to ensure real-time visualization. Thus, acceleration approaches have conventionally been limited to simple methods, such as parallel imaging. Several studies have aimed to reconstruct highly undersampled non-Cartesian real-time data with low latency, thereby enhancing real-time visual tracking during catheterization. Jaubert et al. [35] demonstrated the feasibility of combining an accelerated interactive radial balanced steady-state-free precession sequence with low-latency ML artifact suppression. The results indicated successful real-time suppression of artifacts, indicating that this approach could work effectively without requiring further training on images acquired with varying resolutions or field of view sizes while also being compatible with a wide range of MR catheters.

#### Three-dimensional imaging

AI finds another area of application in cases requiring high-resolution data for the 3D evaluation of anatomy. One example is CMRA [36], where the need to reliably visualize the coronary arteries often results in lengthy acquisition times [37]. Fuin et al. [38] proposed a time-efficient method to reconstruct high-quality isotropic CMRA images during free-breathing. Küstner et al. [39] introduced a super-resolution DL framework to allow free-breathing acquisition in less than a minute. Using a generative adversarial network, this approach facilitated high-resolution isotropic CMRA reconstruction, achieving a 16-fold increase in spatial resolution from low-resolution images. Comparable qualitative a quantitative image quality was obtained within a brief scan time of less than a minute.

Another important use of 3D imaging is in the evaluation of congenital heart disease. Steeden et al. [40] aimed to reduce the scan times for 3D whole-heart images acquired in congenital heart disease patients using a DL single volume super-resolution reconstruction. The prospectively acquired low-resolution acquisition was three times faster than the prospective high-resolution data. Super-resolution reconstruction of the low-resolution data took less than a second per volume and the image quality was improved compared to low-resolution data and closely matching high-resolution data, with sharper edges, fewer residual artifacts and reduced image distortion.

Another study, led by Montalt-Tordera et al. [41], proposed reducing the dose of gadolinium administered for contrast-enhanced CMRA by 80%. An ML network was used to recover characteristics from low-dose images, obtaining image quality comparable to the reference high-dose ones, with a comparable vessel diameter. This method showed potential for reducing both cost and associated risk of contrast agent usage.

However, implementing 3D CINE approaches faces several challenges. Limited availability of high-quality data sets due to the early stage of 3D sequences and the need for enhanced robustness are significant barriers. Furthermore, generalizing algorithms to diverse cardiac pathologies remains challenging due to the scarcity of pathology-specific data, primarily sourced from healthy subjects. In addition, computational resources pose a significant hurdle, with processing large multi-cardiac phase volumes demanding high-performance workstations and potentially hindering real-time applications due to prolonged processing times.

## Radiologist side

**Fig. 4:**
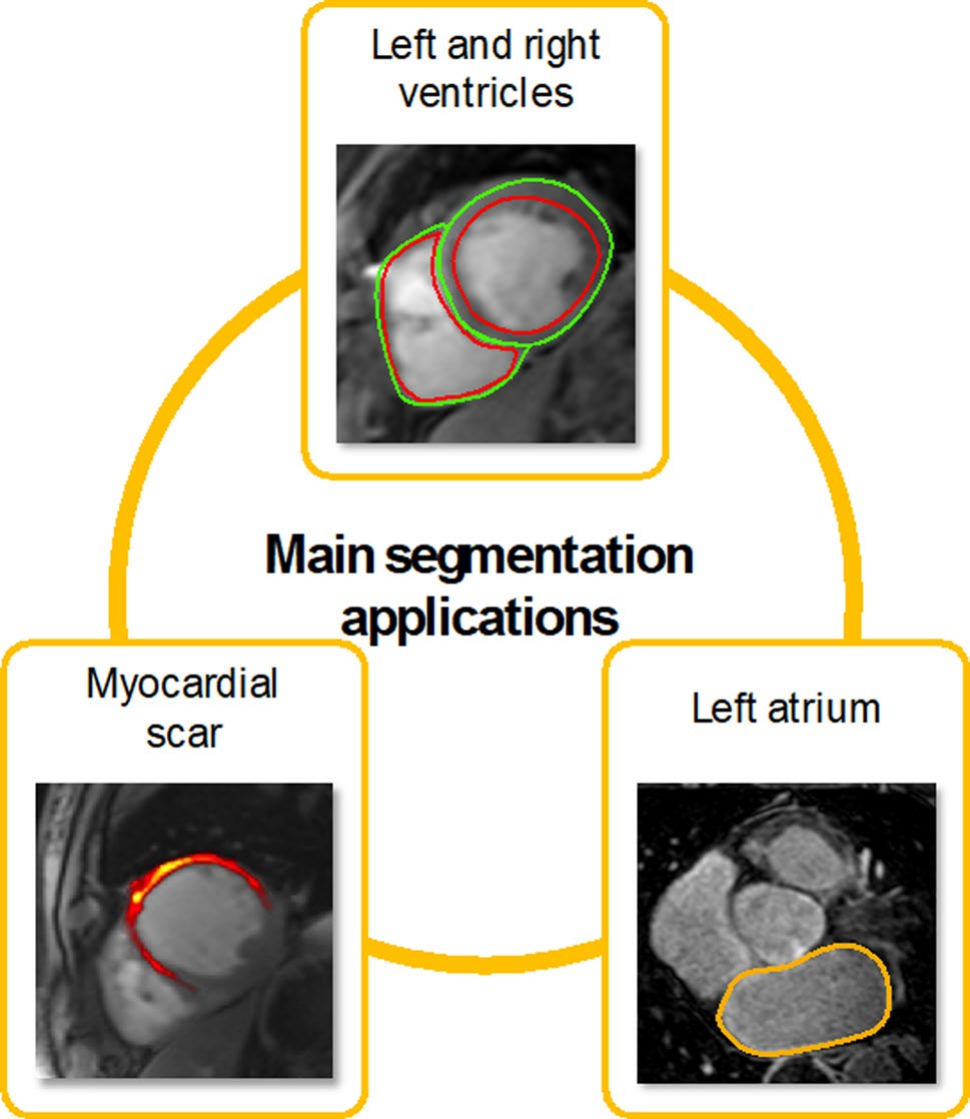
Main cardiac magnetic resonance imaging applications where segmentation is needed. Red: endocardium, green: epicardium. Yellow contour: left atrium

Another significant use of AI in CMR involves enhancing the evaluation of cardiac structures and tissue properties. This field is crucial in clinical cardiology, guiding patient management, aiding in disease diagnosis, assessing risks, and informing therapeutic decisions [42, 43]. However, accurate evaluation requires precise delineation of borders of the chambers and myocardium (a process known as segmentation), as well as vascular structures (Fig. 4). In clinical practice, despite the image acquisition being standardized and being quickly delivered, the image analysis process can be time-consuming, requiring detailed analysis by a clinician [44] that is susceptible to intra- and inter-observer variability [45].

A large body of research has been dedicated to developing automated CMR segmentation methods [46], but for now manual corrections are still needed in the areas where there are lots of trabeculae, the LV outflow tract, apical slices, as well the right ventricle. DL methods have revolutionized medical diagnostic and prognostic tasks [47], outperforming traditional approaches and gaining more popularity in research [48]. Their capacity to automatically discern high-level data features is crucial for object detection. However, DL models are data-driven, relying on a significant amount of annotated data with sufficient variation in relevant image factors for training.

In general, DL-based fully automated cardiovascular segmentations are highly accurate with most of the developed methods [49] achieving very high Dice similarity coefficients. This trend has spurred the emergence of commercially available CMR software tools integrating AI for image analysis, which are already impacting current practice by offering unprecedented speed, accuracy, and versatility. For instance, Circle CVI42® (Calgary, Canada) uses AI-based contouring for quantifying cardiac function, flow, and tissue abnormalities. Similarly, Medviso's Segment CMR (Lund, Sweden) or CASIS (Quetigny, France) use ML and DL algorithms to obtain automatic quantitative and functional analysis from CMR images, enhancing both accuracy and speed of results. Syngo.via, on the other hand, is the dedicated multimodality imaging software solution offered by Siemens Healthineers (Forchheim, Germany), including automatic contouring of organs by incorporating AI-enabled features. All of these software tools cater to clinicians' needs, prioritizing time-saving for enhanced workflow. These examples underscore the significant impact of AI in CMR software tools, streamlining processes, enhancing accuracy, and ultimately improving patient care.

### Ventricles segmentation

**Fig. 5:**
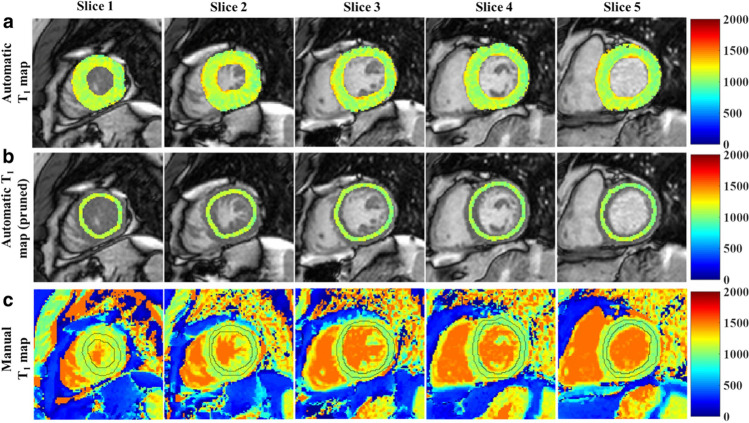
Fully automated analysis of cardiac T1 mapping (adapted from Fahmy et al. [53])

DL frameworks designed for general image segmentation can be directly employed in the context of myocardium and cardiac chamber segmentation within CMR images through pixel-based classification. CNNs are the predominant architectures utilized for short-axis CMR image segmentation, obtaining excellent agreement for commonly-used physiological measures [50]. Early attempts, such as those by Tran [51], used a fully convolutional neural network (FCN) to segment the LV, myocardium, and RV on short-axis MR images. This approach demonstrated superior segmentation performance compared to traditional methods in terms of speed and accuracy. Another example is that of Bai et al. who proposed a fully convolutional approach with a simplified upsampling path, successfully applying it for pixelwise segmentation of 4-chamber, 2-chamber, and short-axis CMR images within seconds [52]. Other work focused on refining segmentation by optimizing network structures for enhanced feature learning capacity [53, 54] (Fig. 5).

Although ventricular segmentation is a 3D problem, most approaches have relied on 2D networks due to low through-plane resolution of multi-slice acquisition and interslice misalignment [55]. The drawback of using 2D networks for cardiac segmentation is that they do not exploit inter-slice dependencies, which is evident in challenging slices, such as apical and basal slices, where ventricle contours are less defined [49]. Efforts have been made to overcome this limitation by introducing additional contextual information to guide 2D approaches, such as shape priors learned from labels or multi-view images [56]. Alternatively, some methods extract spatial information from adjacent slices to aid segmentation, employing recurrent units or multi-slice networks [57].

As mentioned earlier, DL methods can also be used to calculate functional parameters from imaging, such as fully automated determination of LV EF, which can be used to classify patients into different disease categories using hand-crafted features. Puyol-Antón et al. [58] used a database of CMR and cardiac ultrasonography images, along with clinical information, to design an ML-based diagnostic algorithm that can fully automatically identify patients with dilated cardiomyopathy using a support vector machine. In another study involving patients with dilated cardiomyopathy, principal component analysis was applied to ventricular geometric models. The derived shape-based features were integrated into a score independently associated with composite ventricular arrhythmia and sudden cardiac death [59].

Considering this, multi-task learning has also been explored to perform auxiliary tasks that are relevant to the main segmentation task, such as estimation of cardiac function [60]. With this approach, both LV segmentation and cardiac indices estimation can be performed simultaneously, such that these related tasks regularize the network, hence improving the network generalization performance and prediction accuracy. Li et al. [61] introduced an accurate and efficient DL segmentation and regression unified network to segment and quantify the LV simultaneously. The segmentation module leverages a U-Net like 3D transformer model to predict the contour of three anatomy structures, while the regression module learned spatial–temporal representations from the original images and the reconstruct feature maps from segmentation path to estimate the finally desired quantification metrics.

Other recent advancements suggest a growing interest in the utilization of neural networks within a multi-stage pipeline designed to dissect the segmentation problem into smaller subtasks. For instance, Vigneault et al. [62] introduced a network comprising of a U-Net for cardiac chamber localization, a learnable transformation module for image orientation normalization, and a series of U-Nets for intricate segmentation. In a different approach, Abdeltawab et al. [63] employed a framework that initially accurately localizes the centre point of the LV blood pool using a first FCN architecture. Subsequently, a region of interest (ROI) containing the LV was extracted from all heart sections, and a second FCN was employed to segment the LV cavity and myocardium within the ROI.

Ammar et al. [64] proposed a fully automatic framework for heart health assessment. The suggested end-to-end pipeline was constructed on a U-Net convolutional neural network variant for segmentation and a classifier ensemble for disease class prediction. The reported results indicate a mean Dice overlap coefficient of 0.92 for the entire cardiac structure. Similarly, another study used a DL-based approach reliably identifies patients with cardiac amyloidosis with a high degree of accuracy [65]. Conversely, Ghadimi et al. [66] developed a pipeline for the fully automated analysis of cine DENSE data using four CNNs. These networks were tasked with LV segmentation, phase unwrapping, and identification of the anterior RV-LV insertion point for short-axis cine DENSE images. This approach enables fully automatic global and segmental DENSE strain analysis, demonstrating excellent agreement with conventional user-assisted methods. Such advancements may promote increased clinical utilization of DENSE for assessing global and segmental strain in patients with cardiac disease.

Another region of study involves combining neural networks with classical segmentation approaches [67]. This type of pipeline can include segmentation, landmark localization, and atlas propagation [68]. For instance, a study proposed a combination of a biventricular model initialization, a deep learning neural network, and a 3D active shape model segmentation that achieved a fully automated ventricle segmentation with outstanding performance [69].

A significant obstacle faced by deep learning models is the limited availability of training samples. To address this issue, synthetic images offer a simulated alternative. In the realm of CMR, Davies et al. [70] emphasized the efficacy of synthetic images generated through a conditional synthesis framework, especially in situations where data is scarce or absent. These synthetic images played a crucial role in improving the training of a deep learning-based method designed for the segmentation of heart cavities from short-axis CMR images. The resulting algorithm achieved fully automated measurements of LV structure and global systolic function, outperforming human capabilities in terms of both speed and precision.

### Atrial segmentation

The robust delineation of atrial anatomy is important for pre-operative planning of electrophysiological ablation and for scar segmentation and quantification of atrial fibrosis from LGE images. Conventional methods heavily rely on effective initialization and pre-processing techniques, impeding their widespread adoption in clinical applications. The challenges persist when directly segmenting and analysing atrial LGE-MRIs due to variations in intensities resulting from enhanced fibrotic tissue, imaging artifacts, and varying image quality.

As mentioned earlier, current practices for medical image segmentation and 3D reconstruction often rely on labour-intensive manual or semi-automatic methods [71]. In an effort to address this, Preetha et al. [72] employed a 2D FCN to systematically segment the atrium from 3D LGE images, optimizing the network structure for improved feature learning, achieving a mean Dice score of 0.89. Xia et al. [73] proposed an entirely automated two-stage segmentation framework. The first stage uses a 3D U-Net to roughly identify the atrial center from down-sampled images, followed by a second 3D U-Net to precisely segment the atrium in the cropped sections of the original images at full resolution. Their multi-stage approach demonstrated promising results, with a mean Dice score of 0.93 on a test set of 54 cases.

To further enhance the learning of discriminative features and abnormalities from the intricate structure of the left atrium (LA) and pulmonary veins, content-aware networks have incorporated multi-scale convolutions and attention units into the U-Net architecture [74, 75]. This integration aims to improve the network’s capability to discern intricate details and irregularities in the imaging data.

### Tissue characterization

AI has found extensive applications in various tasks related to characterizing myocardial tissue. Specifically, in the identification and quantification of myocardial scar from LGE CMR, ML proves valuable by eliminating the need for subjective, time-consuming, and labour-intensive manual delineation currently employed in routine clinical practice. Scar quantification is a biomarker with intrinsic prognostic information in clinical practice [76]. However, the accuracy and reproducibility of current LGE quantification techniques remain significant challenges.

Numerous DL approaches have been integrated with traditional segmentation methods for scar segmentation. Yang et al. [77] utilized an atlas-based method to identify the left atrium and then applied deep neural networks to detect fibrotic tissue in that region. Popescu et al. [78] developed and validated a deep neural network for automatic, anatomically accurate expert-level scar/fibrosis segmentation from myocardial LGE images. Predicted scar segmentations achieved a 0.57 Dice score when compared to trained expert segmentations. Another study used a CNN-based method for the segmentation of LV myocardial scar from 3DLGE-MR images which reported an average Dice score of 0.94 [79].

In the work of Fahmy et al. [80], a U-Net based network was applied to simultaneously segment the myocardium and the scars from LGE images obtained from patients with hypertrophic cardiomyopathy, achieving rapid segmentation. However, the reported segmentation accuracy for the scar regions was relatively low. Fully-automated scar segmentation remains a challenging task due to kinematic variabilities and abnormalities in contrast-enhanced images caused by infarcted regions in patients.

Furthermore, a novel approach for infarction assessment is being studied by exploring the potential of non-contrast cine-CMR images as an alternative to LGE-CMR images to assess myocardial infarction location and size without gadolinium injection [81, 82]. Xu et al. [83]used a Recurrent Neural Network (RNN) leveraging motion patterns to automatically delineate myocardial infarction areas from cine MR image sequences without contrast agents. This method achieved a high overall Dice score of 0.90 compared to manual annotations on LGE MR images.

## Discovery side

AI is assuming an important role in emerging fields of study where a great number of features need to be extracted from medical images. Through AI-driven algorithms, deeper insights into tissue characteristics and disease progression are attained, providing clinicians with invaluable tools for patient-specific therapy optimization and decision-making.

### Radiomics and texture analysis

**Fig. 6:**
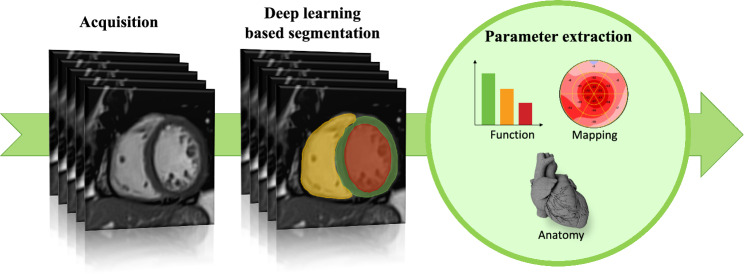
Example of different steps needed in parameter extraction for cardiac magnetic resonance imaging. From left to right: cardiac images are acquired, followed by automated segmentation of various anatomical heart structures, enabling subsequent parameter extraction

The development of ML techniques has dramatically altered the way high-dimensional data can be handled and analysed, which has led to the rise of the expanding field of radiomics. In the medical imaging field, radiomics involves the extraction of a wide range of quantitative features from medical images (Fig. 6), effectively converting them into a data pool that can be mined using AI tools for insights [84]. These features that can be extracted cover a wide spectrum, including morphological, intensity-based, fractal-based, and texture features [85]. In this context, texture analysis (TA) is a subset of radiomics that specifically focuses on quantifying texture features within an image. Its primary objective is to scrutinize and delineate texture patterns within medical images, making it particularly valuable for discerning tissue heterogeneity and distinguishing between different tissue types. Employing various machine learning algorithms, TA can quantitatively assess the spatial variability, patterns, and relationships among neighbouring pixels. This ultimately enables the calculation of advanced imaging metrics, which can transcend the limitations of purely visual image interpretation [86].

Notably, texture features extracted from CMR imaging have shown considerable potential for further exploration and clinical integration [87, 88]. Given the ongoing significance and complexity of characterizing myocardial tissue to differentiate among various cardiac diseases, applying radiomics to CMR imaging data presents a promising avenue for gaining deeper insights into the complex tissue changes and pathologies associated with cardiovascular conditions. In this context, ML models can be used to find novel predictors and implement new approaches using features that are typically unrecognizable to humans [89, 90].

Early applications of radiomics and TA in CMR primarily focused on segmenting scarred tissue areas in cases of myocardial infarction [91]. This segmentation improved the visualization of scarred myocardial tissue and allowed for the extraction of valuable information regarding the characteristics of the underlying myocardial tissue. Subsequently, multiple studies have demonstrated the feasibility of using TA to distinguish between acute and chronic infarctions [92], either by using a combination of non-contrast cine and LGE imaging [93] or cine imaging alone [94]. Several studies have applied texture analysis to LGE segmentation extracting features beyond quantitative scar burden, such as measures of heterogeneity and shape, which have proven valuable in conventional workflows [95, 96].

In addition to infarction, recent reports have shed light on additional applications for these techniques. Several smaller scale studies have demonstrated the potential of texture features to differentiate among various causes of myocardial hypertrophy, including hypertrophic cardiomyopathy, amyloidosis, and aortic stenosis, as well as healthy control subjects [97].

A multitude of extracted features and various ML models have been evaluated for this purpose. For example, a recent study employed an unsupervised deep learning approach on cine CMR among patients with ischemic cardiomyopathy to derive cardiac features. These features were then utilized as inputs in a separate deep neural network, successfully predicting the risk of ventricular arrhythmias [98].

The inverse approach, involving ML in generating predefined features, has also gained recognition in recent literature. For instance, ML-derived measures based on LGE, such as scar complexity, were associated with VA risk in a cohort where entropy proved not to be a significant predictor [99]. A study presented a model based on cine imaging to help identify patients with high risk of fibrosis and screen out patients without fibrosis to avoid unnecessary injection of contrast [100]. Recent studies have used radiomics to characterize and quantify subtle tissue alterations of the ventricular myocardium [101]. One study reported excellent diagnostic accuracy when applying radiomics on T1 and T2 mapping in a cohort of patients with biopsy-proven acute infarct-like myocarditis [102].

Radiomics and texture analysis are opening new possibilities in the field of medical imaging by providing extensive quantitative insights that go beyond what is visually perceptible. These advanced techniques have the potential to improve disease characterisation, treatment planning, and patient outcomes, particularly in the domain of cardiovascular medicine. However, they still face some potential barriers. First, rigorous validation studies in diverse patient populations are essential to demonstrate the reliability, accuracy, and clinical utility of these tools. In addition, seamless integration into existing clinical workflows and compatibility with various MRI systems are crucial for widespread adoption. Furthermore, technical challenges, such as variability in image quality and artifact recognition, also pose significant hurdles for AI algorithms to achieve consistent performance across different imaging scenarios.

### Digital twin

Another promising use of AI has been in precision medicine. Studies have shown the power of patient-specific image-based modelling and simulation for therapy guidance, biomarkers interpretation and patient phenotypic variability interpretation [103–105]. This technological advancement has paved the way for realizing the concept of the Digital Twin in healthcare, representing a comprehensive virtual tool that dynamically integrates an individual's clinical data over time through mechanistic and statistical models [106, 107]. Acting as a digital replica, the Digital Twin is generated by modelling the state of a physical system, collecting data through sensors, and translating this data into a digital format. It acts as a crucial bridge between the physical and digital realms, providing insights into past and present processes and making predictions for the future. This transformative approach is gradually shifting healthcare systems from describing diseases to predicting responses, optimizing treatment selection based on the patient's future state. Creating cardiac digital twins involves developing novel methodologies to analyse patient-specific properties from clinical test data, such as electrocardiography and CMR.

In the context of cardiovascular applications, several studies are employing advanced techniques in medical imaging and computational modelling. One investigation focused on a shape-driven ML model to accurately estimate 3D computational fluid dynamics flow fields in aortas with challenging shapes [108]. Another study explored the utility of biventricular strains derived from model-to-image registration in pulmonary arterial hypertension patients, comparing them to controls [109]. Furthermore, a study calibrated non-invasively cardiac digital twins in healthy subjects using synthetic epicardial activation maps and ECG recordings, with potential applications in daily clinical practice, including adapting to pathological data [110]. Adding to these advancements, a separate study introduced automatic pipelines to generate digital replicas of aortic stenosis cases from clinical CT images, facilitating the simulation of interventional procedures and prediction of outcomes [111].

In summary, cardiovascular digital twin research demonstrates proof-of-concept studies illustrating the application of data-driven approaches aligned with the goals of precision medicine. While the active use of a digital twin for clinical decision-making remains a futuristic prospect [112], early components of the digital twin are already making a clinical impact in improving data acquisition, diagnosis, and therapy planning within the stages of a generic clinical workflow.

## Conclusion

The incorporation of AI in CMR and other imaging techniques has a lot of potential for transforming both research and clinical practice. AI algorithms can equip clinicians with advanced tools for precise diagnosis, personalized treatment planning, and prognostic assessment, which would ultimately lead to improved patient outcomes and enhanced healthcare delivery. As AI continues to advance and evolve, its role in CMR is expected to increase, paving the path for innovation and new changes in cardiovascular medicine.
